# Pelleted Bone Marrow Derived Mesenchymal Stem Cells Are Better Protected from the Deleterious Effects of Arthroscopic Heat Shock

**DOI:** 10.3389/fphys.2016.00180

**Published:** 2016-05-24

**Authors:** Gauthaman Kalamegam, Mohammed Abbas, Mamdooh Gari, Haneen Alsehli, Roaa Kadam, Mohammed Alkaff, Adeel Chaudhary, Mohammed Al-Qahtani, Adel Abuzenadah, Wael Kafienah, Ali Mobasheri

**Affiliations:** ^1^Center of Excellence in Genomic Medicine Research, King Abdulaziz UniversityJeddah, Saudi Arabia; ^2^Sheik Salem Bin Mahfouz Scientific Chair for Treatment of Osteoarthritis by Stem Cells, King Abdulaziz UniversityJeddah, Saudi Arabia; ^3^Department of Orthopedic Surgery, Faculty of Medicine, King Abdulaziz University HospitalJeddah, Saudi Arabia; ^4^Department of Medical Laboratory Technology, Faculty of Applied Medical Sciences, King Abdulaziz UniversityJeddah, Saudi Arabia; ^5^Faculty of Applied Medical Sciences, Center of Innovation in Personalized Medicine, King Abdulaziz UniversityJeddah, Saudi Arabia; ^6^School of Cellular and Molecular Medicine, University of BristolBristol, UK; ^7^The D-BOARD European Consortium for Biomarker Discovery, The APPROACH Innovative Medicines Initiative Consortium, Faculty of Health and Medical Sciences, University of SurreySurrey, UK; ^8^Arthritis Research UK Centre for Sport, Exercise and Osteoarthritis, Arthritis Research UK Pain Centre, Medical Research Council and Arthritis Research UK Centre for Musculoskeletal Aging Research, University of Nottingham, Queen's Medical CentreNottingham, UK

**Keywords:** mesenchymal stem cells, osteoarthritis (OA), cell viability, population doubling, fluorescence activated cell sorting (FACS), differentiation, heat shock

## Abstract

**Introduction:** The impact of arthroscopic temperature on joint tissues is poorly understood and it is not known how mesenchymal stem cells (MSCs) respond to the effects of heat generated by the device during the process of arthroscopy assisted experimental cell-based therapy. In the present study, we isolated and phenotypically characterized human bone marrow mesenchymal stem cells (hBMMSCs) from osteoarthritis (OA) patients, and evaluated the effect of arthroscopic heat on cells in suspension and pellet cultures.

**Methods:** Primary cultures of hBMMSCs were isolated from bone marrow aspirates of OA patients and cultured using DMEM supplemented with 10% FBS and characterized for their stemness. hBMMSCs (1 × 10^6^ cells) cultured as single cell suspensions or cell pellets were exposed to an illuminated arthroscope for 10, 20, or 30 min. This was followed by analysis of cellular proliferation and heat shock related gene expression.

**Results:** hBMMSCs were viable and exhibited population doubling, short spindle morphology, MSC related CD surface markers expression and tri-lineage differentiation into adipocytes, chondrocytes and osteoblasts. Chondrogenic and osteogenic differentiation increased collagen production and alkaline phosphatase activity. Exposure of hBMMSCs to an illuminated arthroscope for 10, 20, or 30 min for 72 h decreased metabolic activity of the cells in suspensions (63.27% at 30 min) and increased metabolic activity in cell pellets (62.86% at 10 min and 68.57% at 20 min). hBMMSCs exposed to 37, 45, and 55°C for 120 s demonstrated significant upregulation of BAX, P53, Cyclin A2, Cyclin E1, TNF-α, and HSP70 in cell suspensions compared to cell pellets.

**Conclusions:** hBMMSC cell pellets are better protected from temperature alterations compared to cell suspensions. Transplantation of hBMMSCs as pellets rather than as cell suspensions to the cartilage defect site would therefore support their viability and may aid enhanced cartilage regeneration.

## Introduction

Osteoarthritis (OA) is a chronic degenerative disease of load-bearing synovial joints that commonly affects a significant and ever increasing proportion of the aging population. OA is the most common joint disorder in the elderly. Its incidence has been rising steadily and is expected to escalate further with the projected world-wide increase in the aging population, impacting on the existing socio-economic burden (Arden and Nevitt, 2006). Nearly 50 million Americans are already afflicted with OA, with 50% of the individuals affected being more than 65 years of age (Center for Disease Control Prevention (CDC), 2010). By 2030, it is estimated that about 20% of the European and American populations will suffer from OA (De Bari et al., 2010). In the same period in the Middle East, the indicators of aging, namely the standardized prospective median age and the average age of the population are projected to increase from 20.9 and 26.0 in 2010 to 23.5 and 31.4 (Lutz et al., 2008). Furthermore, the cultural prayer position and increased obesity in the Middle Eastern population pose additional risks for the development of OA in the aging population.

OA is characterized by progressive joint degeneration, pain, effusion and limitation of mobility with eventual loss of function (Buckwalter and Lane, 1997). The entire articulating joint together with its constituent peri-articular tissues including bone, ligaments, joint capsule and synovium and are involved in the progression of OA, highlighting the complex nature of this disease (Loeser et al., 2012).

Although multiple factors are implicated in the aetiopathogenesis of OA, the underlying molecular mechanisms still remain unclear. Several hypotheses have been proposed to account for the development of OA including age-related “wear and tear,” chondrocytes' poor response to growth factors, increased sensitivity to pro-inflammatory stimuli, excessive cross-linking and structural modification of collagens leading to alterations in the bio-mechanical properties of articular cartilage, mitochondrial dysfunction, oxidative stress and low-level inflammation (Mobasheri et al., 2013). Fundamentally, the number of chondrocytes in articular cartilage decreases with age resulting in impaired extracellular matrix repair and reduced production of new matrix proteins. In addition the absence of blood vessels and stem cells within the cartilage limits the natural healing capacity (Henrotin and Reginster, 1999; Mobasheri et al., 2013) although evidence suggests that innervation and vascularization increases in the late stages of disease progression.

Non-steroidal anti-inflammatory drugs, opioids, topical formulations, intra-articular injections and nutraceuticals are all used in OA treatment, but they are largely symptom modifying agents and there is little evidence to suggest that any of them have the capacity to act as disease modifying osteoarthritis drugs (DMOADs). Pharmacological management with DMOADs may relieve pain to some degree, but does not offer any structure modification (Reid et al., 2012). Various surgical methods have been developed to restore damaged cartilage and improve joint function. These include microfracture, subchondral drilling, abrasion arthroplasty and autologus chondrocyte implantation (ACI). The aim of these techniques is to promote intrinsic healing by promoting vascular invasion, fibrin clot formation and recruitment of stem cells (Brittberg et al., 1994; Vinatier et al., 2009; Orth et al., 2014). However, the poor biomechanical properties of the new scar tissue in microfracture, as well as donor site morbidity, low cellularity and surrounding cartilage damage at the transplantation site in ACI limit their clinical utility and surgical outcomes (Brittberg et al., 1994; Vinatier et al., 2009; Orth et al., 2014). When pharmacological and surgical management fail, which is so often the case in the clinical setting, the disease progresses to end stage OA, where total joint replacement becomes the only option. However, the life span of currently available prostheses is limited and there is increasing demand for safer and more effective surgical treatments and therapeutic strategies (Kurtz et al., 2007).

Regenerative medicine offers great potential for therapeutic intervention in OA and could provide an excellent alternative to total joint replacement. The use of autologus matrix induced chondrogenesis (AMIC) in OA (Gille et al., 2013) and intra-articular injection of meniscal stem/progenitors cells (Shen et al., 2014) are some recent advances in this area. Autologus and allogeneic stem cells derived from various sources (*viz*. bone marrow, synovium, adipose tissue etc.) have been used for treatment of OA with variable success (Garcia-Alvarez et al., 2011; Mobasheri et al., 2014). These are either directly injected into the damaged site or differentiated into cartilage together with tissue engineered scaffolds or following treatment with growth factors.

The introduction of biological agents such as stem cells into the joint normally requires arthroscopic techniques. Temperature increases of 52.0 and 49.5°C following monopolar and bioplar radiofrequency application with irrigation have been reported during wrist arthroscopy in cadaveric models (Huber et al., 2013). Animal studies have demonstrated ultrastructural changes in the size and cross-sectional diameter of the joint capsular collagen fibrils ranging from 22.5 to 50.4% following increased temperatures of 45 and 85°C (Lopez et al., 1998). Bone drilling, a common treatment for operative fracture also generates heat and temperatures above 47°C are known to cause osteonecrosis (Augustin et al., 2012). The aim of this study was to investigate the impact of temperature changes associated with arthroscopic procedures on cellular activity and/or survival in suspension and pellet cultures of human bone marrow mesenchymal stem cells (hBMMSCs), which are increasingly studied as a promising cell source for cartilage regeneration.

## Materials and methods

### Derivation and propagation of hBMMSCs

Bone marrow aspirates were harvested from the iliac crest of OA patients undergoing surgical treatment in the Department of Orthopedics, King Abdulaziz University Hospital, Jeddah, Kingdom of Saudi Arabia. Prior to sample collection informed patient consent was obtained and the research study was carried out following Institutional Research Ethics Committee approval [11–557]. hBMMSCs were isolated using earlier published protocols (Brady et al., 2014). Briefly, the bone marrow aspirate (5–6 ml) was collected in heparinized tubes (Becton Dickinson, BD) and directly plated into tissue culture flasks (~2 ml of aspirate/T175 cm^2^ flask; Greiner) and cultured using Dulbeccos's modified Eagle's medium (Sigma), supplemented with 10% fetal bovine serum (Sigma), 2 mM Gluta-Max (Life Technologies) and antibiotic solution (penicillin, 100 u/mL; streptomycin 100 μg/mL - Sigma) under standard culture conditions of 37°C and 5% carbon dioxide (CO_2_) in atmospheric air for 5–7 days. The first media change was carried out on day 5 and subsequently every 2 or 3 days until subculture. Dead/suspended cells and cellular debris were washed away with media changes and the hBMMSCs were retained as a consequence of their plastic adherence. Basic fibroblast growth factor (bFGF; Peprotech) at 5 ng/mL was added to the culture medium to facilitate hBMMSCs expansion and early passages of the derived hBMMSCs (< P5) were used in the experiment Expanded cells were frozen using ProFreeze (Lonza) in liquid nitrogen and stored for subsequent use.

### Effect of arthroscope temperature on cell morphology and metabolic activity

Baseline characterization included assessment of cell morphology and survival. The hBMMSCs were seeded in 24 well tissue culture plates at 2 × 10^4^ cells/well. Cell morphology and their metabolic activity were analyzed using phase contrast microscopy and MTT assay respectively. To study the effect of temperature related changes that may result with use of the arthroscope in a clinical setting hBMMSCs (1 × 10^6^ cells) were used either as cell suspensions or cell pellets. The arthroscope was sterilized and suspended vertically down using a stand with fixed clamp and placed within the biosafety cabinet. The suspension height of the arthroscope from the clamp was kept constant and the illuminated end was placed into the medium (10 mL) containing cell suspensions or cell pellets in 50 mL graduated Falcon tubes. The samples from both cell suspension and cell pellet groups were exposed for 10 min (Group A), 20 min (Group B), or 30 min (Group C). The cell suspensions and pellets were then gently mixed and 2 × 10^4^ cells/well were seeded in a 24 well plate and cultured under standard culture conditions of 37°C in 5% atmospheric air for 72 h and both cell morphology and metabolic activity were assessed.

Cellular activity assays were also performed on cells from all the groups using the MTT kit (3-(4,5-dimethylthiazolyl-2)-2,5-diphenyltetrazolium bromide; Sigma). Briefly, 10 μL MTT reagent (0.5 mg/mL) was added to cultures, incubated for 4 h followed by medium removal and addition of 200 μL solubilization reagent. Culture plates were further incubated for 2 h and absorbance at 570 nm (ref 650 nm) was measured using a spectrophotometer (μQuant; BioTek).

### CD marker analysis

Cultures of hBMMSCs were analyzed for expression of MSC related cluster of differentiation (CD) markers. Briefly, monolayer cultures of hBMMSCs were dissociated using 0.25% Trypsin-EDTA (Life Technologies) for 3 min. Trypsin activity was inhibited by addition of culture medium containing 10% fetal bovine serum (FBS). The cell suspension was centrifuged at 300 g × 5 min and the cell pellet was then resuspended in phosphate buffered saline without calcium and magnesium (PBS-) containing 3% FBS to obtain single cell suspension. Separate aliquots (2 × 10^5^ cells) were used for MSC isotype cocktail (Miltenyi Biotec), MSC phenotyping cocktail (Miltenyi Biotec) or in combination with other primary monoclonal antibodies (CD44, CD29—BD Pharmingen) to avoid interference with same fluorochromes. The MSC isotype cocktail comprised of fluorochrome conjugated monoclonal antibodies, namely mouse IgG1-FITC, mouse IgG1-PE, mouse IgG1-APC, mouse IgG1-PerCp and mouse IG2a-PerCp. The MSC phenotyping cocktail comprised of both positive (CD73-APC, CD90-FITC, CD105-PE) and negative (CD34/CD45/CD14/CD20-PerCp) fluorochrome conjugated monoclonal antibodies. The cells were incubated with respective antibodies at 1:10 dilution for 15 min at 4°C; then washed with 1 mL of 3% FBS and centrifuged at 300 g × 5 min. The supernatant was discarded and the cells were resuspended in 500 μl of 3% FBS before analysis using a FACS Aria III instrument (BD BioSciences), which is equipped with a 488 nM (blue) laser and a 561-nM (yellow-green) laser for uncoupled excitation and detection of FITC and PE fluorochromes. In addition to increasing the sensitivity of PE detection, this set-up eliminated the PE-FITC spill over, thereby eliminating the need for compensation. As an additional measure, single-stained control tubes for each color was analyzed to rule out the need for compensation as well as set up the detection range for each fluorochrome.

### Trypan blue viability/population doubling time

The hBMMSCs were seeded at 2 × 10^4^ cells/well in a 24 well plate and cultured for up to 3 days to determine cell viability and population doubling time (PDT). The cells were trypsinized daily at the same time and the live/dead cell counts were obtained following trypan blue vital staining. Three replicates were carried out for each sample. PDT (http://www.doubling-time.com) was calculated using the formula:

$$\begin{array}{l} \text{Doubling time}=\\ \;\frac{\text{Duration}{\;}^{*}\;\log\left(2\right)}{\text{Log}\left(\text{final concentration}\right)-\text{Log}\left(\text{initial concentration}\right)} \end{array}$$

### hBMMSCs differentiation into adipocytes

The hBMMSCs (2 × 10^4^ cells/well) were seeded into 24 well plates and allowed to reach confluence before being stimulated to differentiate using the StemPro adipocyte differentiation kit (A10070-01, ThermoFisher Scientific). Control cells were cultured using the differentiation basal medium alone while cells stimulated to undergo adipocyte differentiation were cultured in basal medium fortified with adipocytic supplement (StemPro®) for up to 21 days with fresh media change every 3–4 days. Following differentiation, the cells were fixed in 4% formaldehyde solution for 30 min, rinsed twice with PBS and stained with oil Red O (Sigma) to visualize lipid vacuoles.

### hBMMSCs differentiation into osteoblasts

The hBMMSCs (2 × 10^4^ cells/ well) were seeded into 24 well plates and stimulated to differentiate along the osteoblastic lineage using (StemPro ®) osteoblast differentiation kit (A10072-01, ThermoFisher Scientific). The control cells were cultured using the differentiation basal medium alone while the cells stimulated to undergo osteoblastic differentiation were cultured in basal medium fortified with osteogenic supplement (StemPro®) for up to 21 days with fresh media change every 3–4 days. Following differentiation, the cells were fixed in 4% formaldehyde solution for 30 min, rinsed twice with PBS and stained with Alizarin red (Sigma) solution, washed and analyzed by light microscopy.

### hBMMSCs differentiation into chondrocytes

The hBMMSCs (2 × 10^4^ cells/ well) were seeded into 24 well plates and stimulated to differentiate along the chondrocytic lineage using (StemPro ®) chondrocyte differentiation kit (A10071-01, ThermoFisher Scientific). The control cells were cultured using the differentiation basal medium alone while the cells stimulated to undergo chondrocyte differentiation were cultured in basal medium with chondrogenic supplement (StemPro®) for up to 21 days with fresh media change every 3–4 days. Following differentiation, the cells were fixed in 4% formaldehyde solution for 30 min, rinsed twice with PBS and stained with Alcian blue solution prepared in 0.1 N HCl, washed and analyzed by light microscopy.

### Collagen (sircol) assay

The secreted total collagen levels from both chondrocyte control and differentiation cultures were evaluated using the Sircol™ (chemical collagen assay) kit (Bioclor) according to the manufacturer's instructions. Briefly, 1 ml of the Sircol reagent was added to the 100 μl of the standards and samples (1:20, diluted in distilled water) in 1.5 ml microcentrifuge tubes (Eppendorf); mixed well and placed on a mechanical shaker for 30 min to enable collagen-dye complex precipitation. The contents were then centrifuged at 12,000 rpm for 10 min and the supernatant was carefully decanted taking care to avoid loss of cell pellets. The unbound dye was removed by layering 750 μl of ice-cold acid-salt wash reagent (Kit content) followed by centrifugation at 12,000 rpm for 10 min. The supernatant was carefully removed and 250 μl of alkali reagent (kit content) was added and vortexed to dissolve the bound dye. Absorbance at 555 nm was spectrophotometrically measured using a microplate ELISA reader (μQuant-BioTek) and the collagen concentration was determined.

### Alkaline phosphatase assay

Alkaline phosphatase (ALP) activity levels from both control and differentiated osteoblast cultures were evaluated by measuring the release of p-nitrophenylphosphate (Sigma) as reported earlier (Gauthaman et al., 2011). Absorbance at 555 nm was spectrophotometrically measured using a microplate ELISA reader (μQuant-BioTek) and ALP concentration was determined.

### Quantitative real-time polymerase chain reaction (QRTPCR)

The hBMMSCs (2 × 10^6^ cells) either as pellets or cell suspensions in 500 μl PBS, were subjected to heat shock for various temperatures (37, 45, and 55°C) for 120 s using the hotplate method as reported earlier (Dolan et al., 2012) as the temperature was identified to be more accurate. Total RNA was then extracted from the heat shock treated cells using QiagenTM RNA extraction kit reagent (Invitrogen, Life Technologies). RNA quality and quantity were measured using a NanodropTM spectrophotometer (Nanodrop technologies, Wilmington, DW) and all samples were treated with DNase-I prior to first strand cDNA synthesis with random hexamers using the SuperScript™ first strand synthesis system (Invitrogen). Primer sequences were taken from earlier published studies (Alekseenko et al., 2014; Liang et al., 2015) and the details are given in Table 1. QRT-PCR analysis was performed with the ABI StepOne Plus Real-Time PCR System (Applied Biosystems, Foster City, CA) using SYBR green and relative quantitation was performed using the comparative CT (2-ΔΔCT) method.

**Table 1:** **The genes and primer sequences used for quantitative real time PCR**.

**Genes**	**Primer sequences**
GAPDH	F: 5′-ACCACAGTCCATGCCATCAC-3′ R: 5′-TCCACCACCCTGTTGCTGTA-3′
BAX	F: 5′- TGGAGCTGCAGAGGATGATTG -3′ R: 5′- GCTGCCACTCGGAAAAAGAC -3′
BCL2	F: 5′- GGCTGGGATGCCTTTGTG -3′ R: 5′- CAGCCAGGAGAAATCAAACAGA -3′
P53	F: 5′- GCGCACAGAGGAAGAGAATC -3′ R: 5′- CTCTCGGAACATCTCGAAGC -3′
TNF-α	5′-GGT-GCTTGT-TCC-TCA-GCC-TC-3′ 5′-CAG-GCA-GAAGAG-CGT-GGT-G-3′
Cyclin A2	5′-CCT CTC CTC CAT GTC TGT GTT-AAG-3′ 5′-GTG CTC CAT TCT CAG AAC CTG CTT-3′
Cyclin E1	5′-TGC AGA TCG CAG AGC TTC TA-3′ 5′-CTT TCT TTG CTT GGG CTT TG-3′
HSP70	5′-TCTTGGCACCACCTACTCTTG-3′ 5′-CATCACCGATCAACCGTTCAG-3′

*F, Forward primer; R, Reverse primer*.

### Statistical analysis

The differences observed between treated and control cell numbers, collagen content, alkaline phosphatase and gene expression assays were analyzed using the Students *t*-test with the statistical package for Social Sciences (SPSS 13). The results were expressed as mean ± standard error of the mean (SEM) from three different replicates for individual assays and a value of *p* < 0.05 was considered to be statistically significant.

## Results

### Morphology and growth characteristics of hBMMSCs

**Figure 1:**
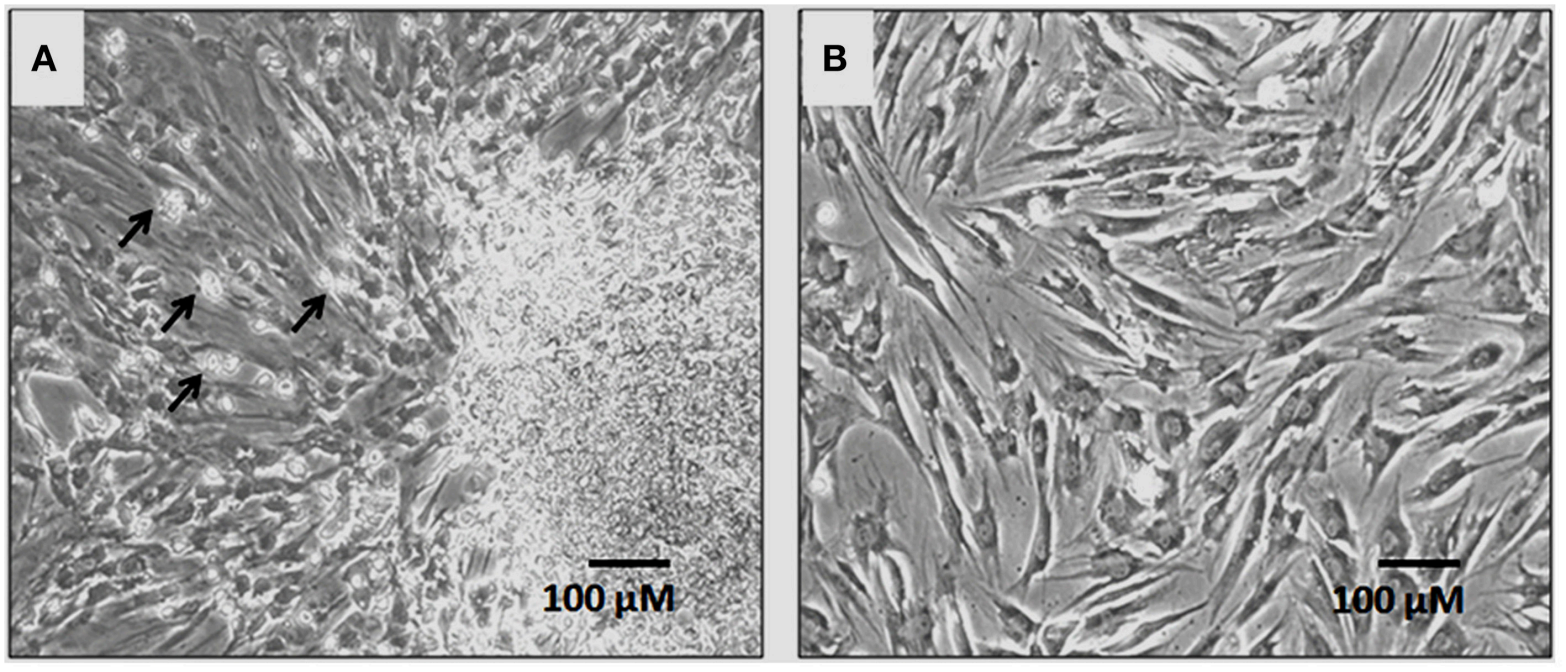
**Phase contrast microscopic images showing primary cultures of human bone marrow derived mesenchymal stem cells (hBMMSCs) at passages P0 (A) and P1 (B)**. Non-adherent cells are indicated by black arrows in P0 **(A)**. The hBM-MSCs at P1 exhibited epitheloid and short spindle shaped and morphology. (Magnification 10X).

In primary cultures by day 5–7 the hBMMSCs adhered to the culture surface as multiple colony forming units (CFU) and the cell numbers continued to expand by day 7–9 reaching up to 60–70% confluence. The non-adherent cells that were present in early cultures were washed away with media changes leaving behind only adherent hBMMSCs. The hBMMSCs derived from the bone marrow aspirate of OA patients showed epitheloid and short spindle shaped cells in early passages (Figure 1). The initial number of cells in primary monolayer cultures varied from 1.4 ± 0.4 × 10^6^ to 1.9 ± 0.6 × 10^6^ cells (from 5 mL bone marrow aspirate cultured in three T175 cm^2^ flasks). However, with subsequent passages where uniform monolayer cultures were obtained, the cell numbers could be expanded to 2.1 ± 0.4 × 10^6^ cells per T175 cm^2^ flask.

### Surface marker characterization of hBMMSCs

**Figure 2:**
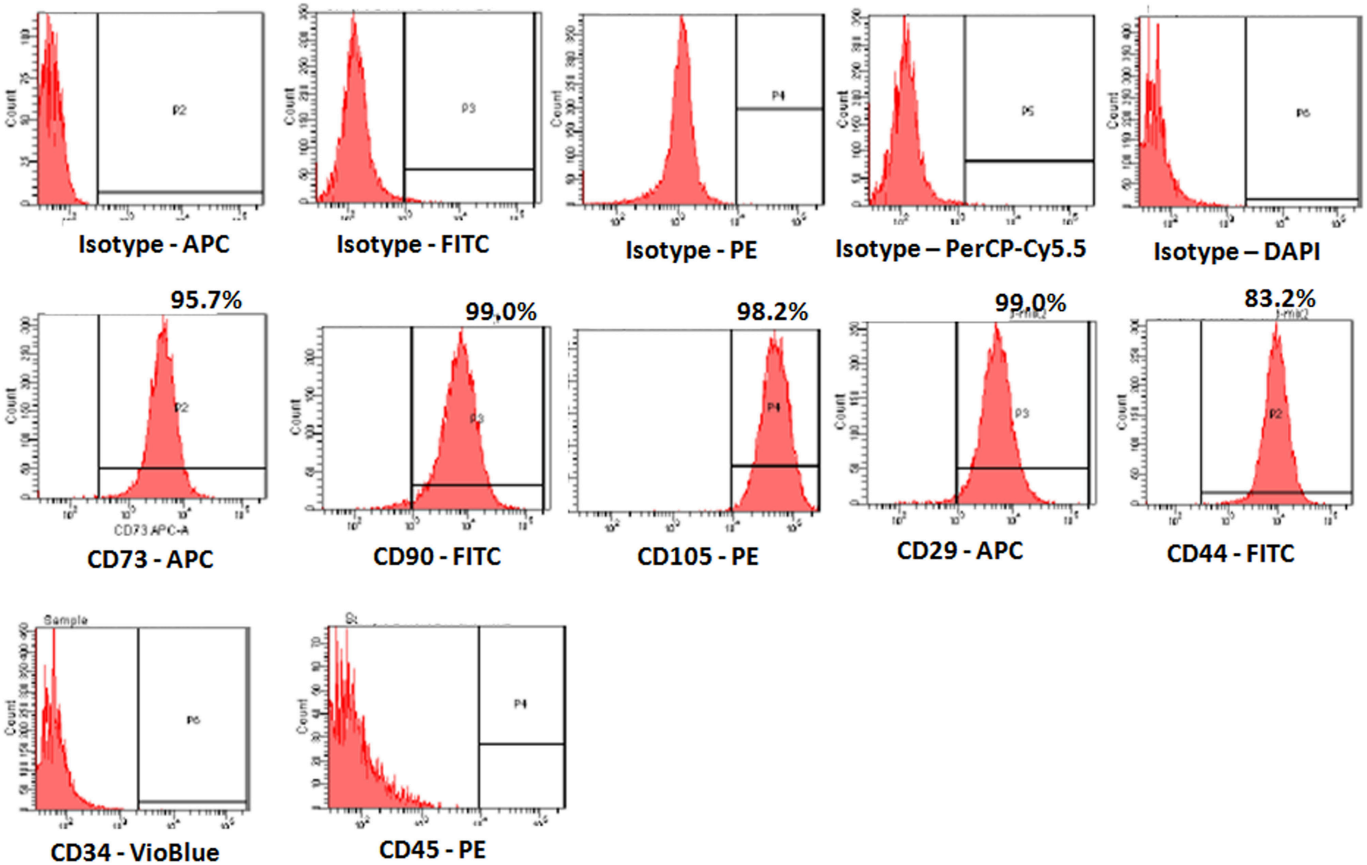
**Representative Fluorescent activated cell-sorting (FACS) analysis showing the CD marker expression pattern in human bone marrow mesenchymal stem cells (hBMMSCs). Top panel:** Respective isotype controls; **Middle panel:** MSC positive CD markers; **Bottom panel:** MSC Negative CD markers.

The derived cells analyzed for CD markers expression demonstrated high percentages of positive MSC related CD markers, namely CD73 (95.7%), CD90 (99.0%), CD105 (98.2%), CD44 (99.0%), and CD29 (83.2%) compared with respective isotype matched controls (Figure 2). These cells were negative for CD34 and CD45, the haematopoietic stem cell related CD markers (Figure 2).

### hBMMSCs population doubling and cell viability

**Figure 3:**
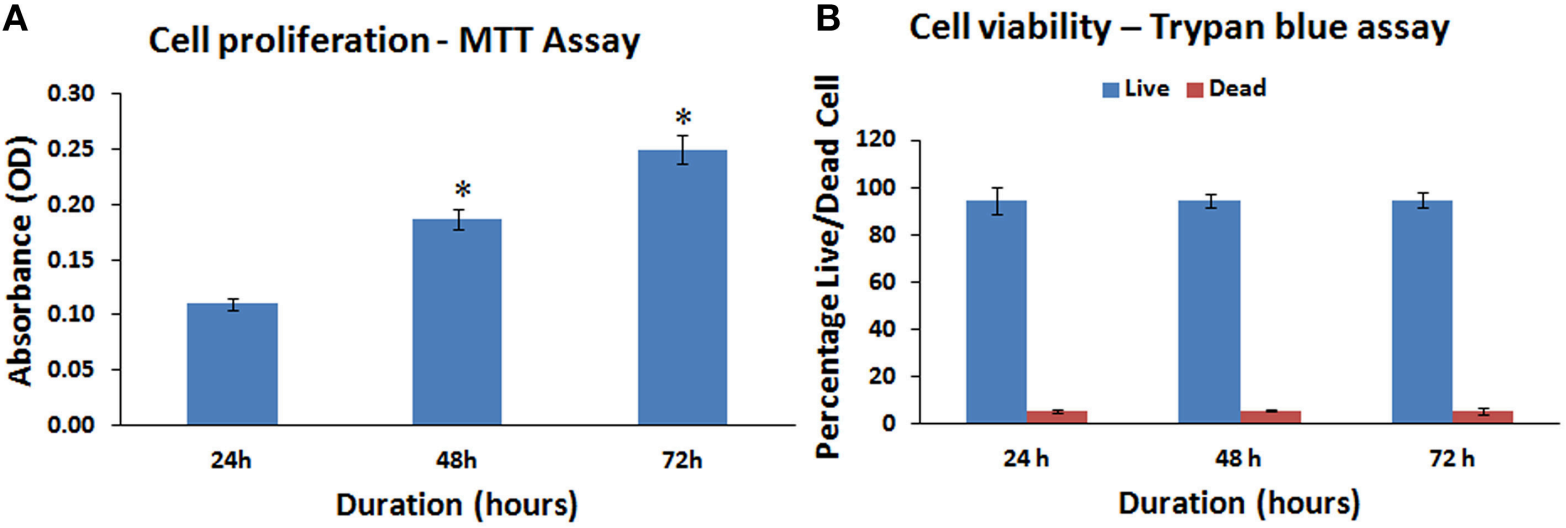
**Mitochondrial activity (MTT) and cell viability (trypan blue) assay of the human bone marrow mesenchymal stem cells (hBMMSCs). (A)** Cellular activity of the hBMMSCs by MTT assay at 24, 48, and 72 h showing increase in cell numbers with increase in time. **(B)** Trypan blue viability assay showing the percentage of live and dead cells at 24, 48, and 72 h. All values are expressed as mean ± standard error of the mean (SEM) from three different samples. Asterisks (*) indicate statistical significance at *p* < 0.05 compared to respective controls.

The hBMMSCs demonstrated a mean increase in cell numbers from 24 to 72 h. There was a mean increase of 72.73 and 127.27% at 48 and 72 h respectively (Figure 3A). These mean increases in cell numbers were statistically significant (*P* < 0.05).

The hBMMSCs showed an increasing linear growth profile over time with every passage and the PDT was 24.33–29.56 h with growth rate 0.0285 and 0.0234 (Growth rate = number of doublings that occur per unit of time) at P1 and P5 respectively. Cell growth were slower with increase in passage number. The trypan blue viability showed that most of the cultured hBMMSCs remained viable in culture platforms that could be used for *in vitro* assays. The percentage of viable cells were 94.57, 94.33, and 94.77% at 24, 48, and 72 h respectively (Figure 3B).

### Differentiation potential of hBMMSCs

**Figure 4:**
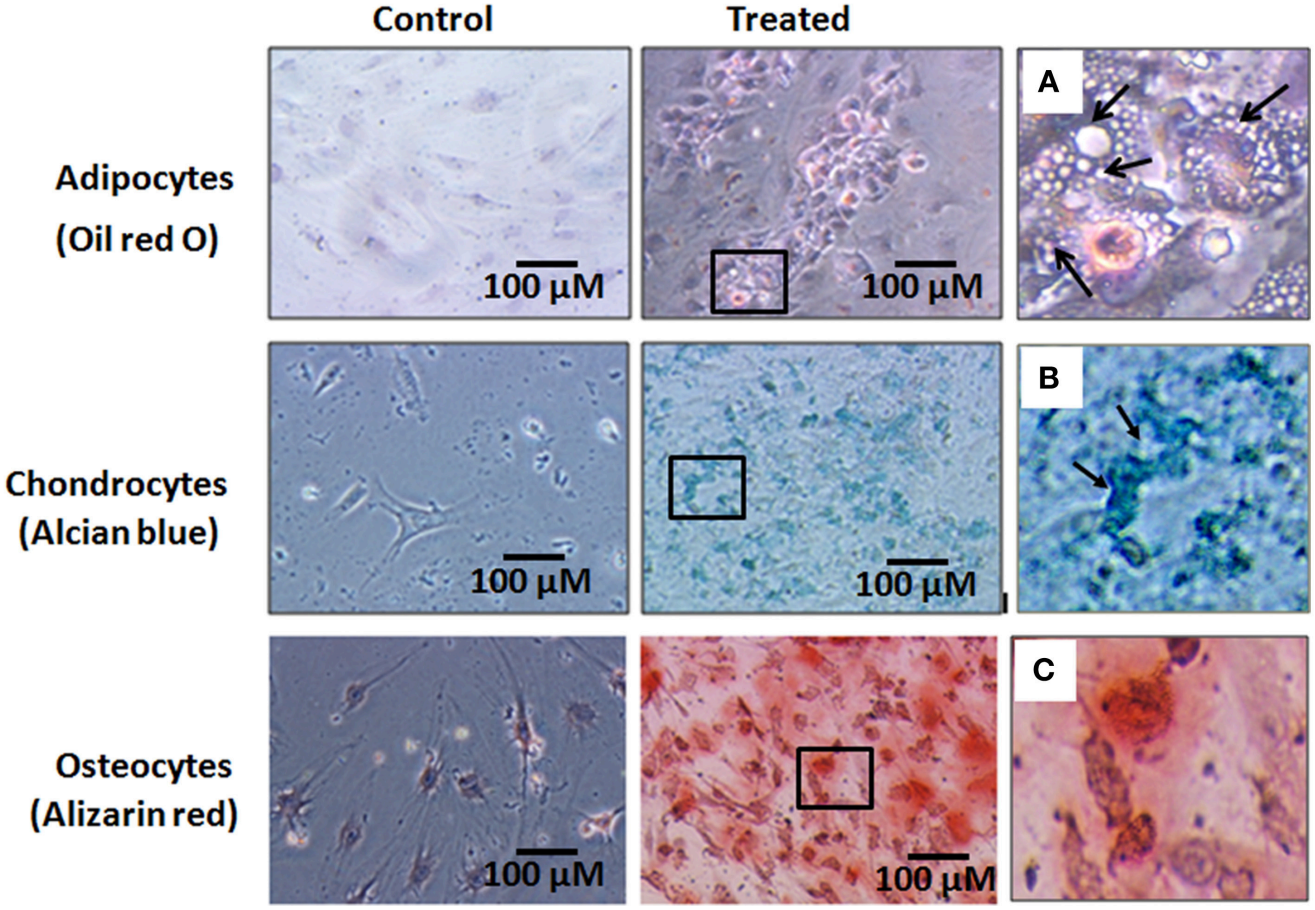
**Histological images of the human bone marrow mesenchymal stem cells (hBMMSCs) differentiated into adipocytes, chondrocytes and osteoblasts and stained with oil red O, Alcian blue and Alizarin red stains respectively**. **(A–C)** are the magnified images of the boxed areas from the treated images of adipocytes, chondrocytes and osteoblasts respectively. Arrows in **(A)** indicate cell vacuolation, and arrows in **(B)** indicate the chondrocyte like cells. Magnification 10X.

The hBMMSCs showed differentiation into adipocytes, chondrocytes and osteoblasts with culture in respective differentiation medium (StemPro®). The cells differentiated along the adipocyte lineage demonstrated lipid vacuolation starting as early as day 14 and the number of cells with lipid vacuoles increased when cultured until 21 days and these cells demonstrated positive staining with oil red O (Figure 4A). hBMMSCs cultured in chondrogenic differentiation medium demonstrated aggregation of cells when cultured for up to 21 days and they included chondrocyte like cells that demonstrated positive staining with Alcian blue compared to the control (Figure 4B). Osteogenic differentiation potential of hBMMSCs cultured in osteogenic differentiation medium showed positive staining with Alizarin red indicative of calcium mineralization (Figure 4C).

### Collagen secretion and alkaline phosphatase activity of differentiated hBMMSCs

**Figure 5:**
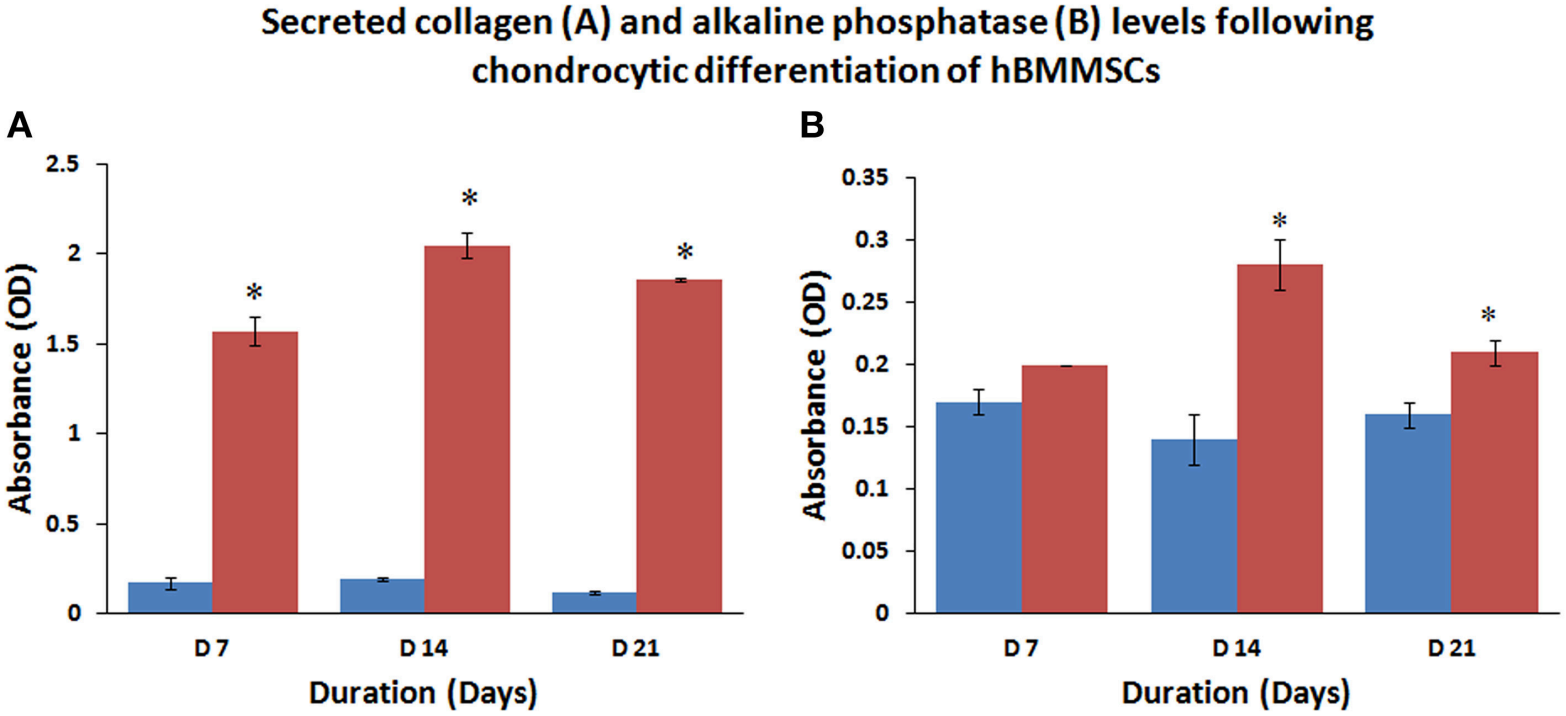
**Collagen secretion (A) and alkaline phosphatase induction (B) increased with time following chondrocytic and osteoblastic differentiation of hBMMSCs compared to control, reflecting the increase in commitment of progenitor cells toward the respective phenotype**. Values are expressed as mean ± standard error of the mean (SEM) from three different samples. Asterisks (*) indicate statistical significance at *p* < 0.05 compared to respective controls.

The amount of secreted collagen measured using the Sircol assay confirmed that chondrocytic differentiating cells secreted large amounts of collagen at various culture periods compared to undifferentiated control cells (Figure 5A). The mean percentage increases in collagen levels were 923.53, 1078.96, and 1550.00% at 7, 14, and 21 days compared to their respective controls and these increases were statistically significant (*P* < 0.05).

The culture media analyzed for alkaline phosphatase activity from control and osteocytic differentiated cells showed increase in alkaline phosphatase levels compared to control undifferentiated cells (Figure 5B). The mean percentage increases in alkaline phosphatase levels were 17.65, 200.10, and 131.25% at 7, 14, and 21 days compared to their respective controls and only those increases at 14 and 21 days were statistically significant (*P* < 0.05).

### Effect of arthroscope temperature on the metabolic activity of hBMMSCs

**Figure 6:**
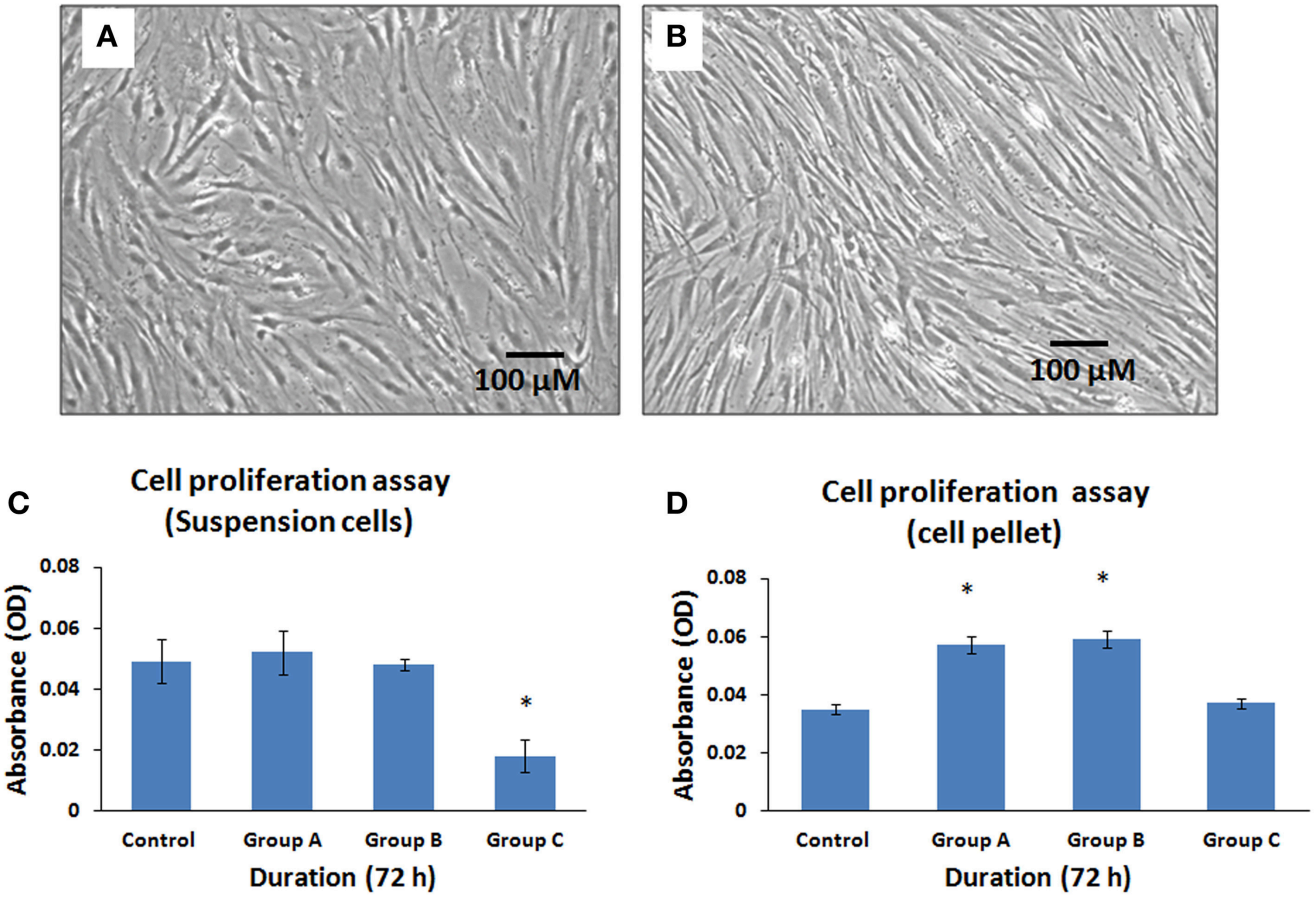
**Phase contrast microscopic images of human bone marrow mesenchymal stem cells (hBMMSCs) following exposure either as cell suspension arm (A) or cell pellet arm (B) to illuminated arthroscopic temperature and cultured for 72 h**. Decrease in cell density was observed in cell suspension arm **(A)** compared to cell pellet arm **(B)**. (Magnification 10X). **(C,D)** Cellular activity assay of the hBMMSCs exposed to an illuminated arthroscope at 72 h, either as cell suspension **(C)** or cell pellet **(D)**. Values are expressed as mean ± standard error of the mean (SEM) from three different samples. Asterisks (*) indicate statistical significance at *p* < 0.05 compared to respective controls.

The cells that were exposed to the illuminated arthroscope for 10 min (Group A), 20 min (Group B), and 30 min (Group C) demonstrated differences in metabolic activity rates for both cell suspension group and cell pellet group when subsequently cultured and assayed for 72 h. There were no changes in cell morphology between suspensions and cell pellets. However, there was a decrease in metabolically active cell numbers in the cell suspensions (Figure 6A) compared to cell pellets (Figure 6B). The cell suspensions displayed a minimal increase of 6.12% and a minimal decrease of 2.04% at 10 and 20 min, respectively, compared to controls (Figure 6C). Maximum mean decrease in cell numbers were observed at 30 min (by 63.27%) compared to the control (Figure 6C) and this was statistically significant (*P* < 0.05). The cell pellet showed mean increases in cell numbers by 62.86, 68.57, and 5.71% at 10, 20, and 30 min respectively, compared to controls (Figure 6D). However, only those increases observed at 10 and 20 min were statistically significant (*P* < 0.05).

### Effect of heat shock on hBMMSCs gene expression (QRTPCR)

**Figure 7:**
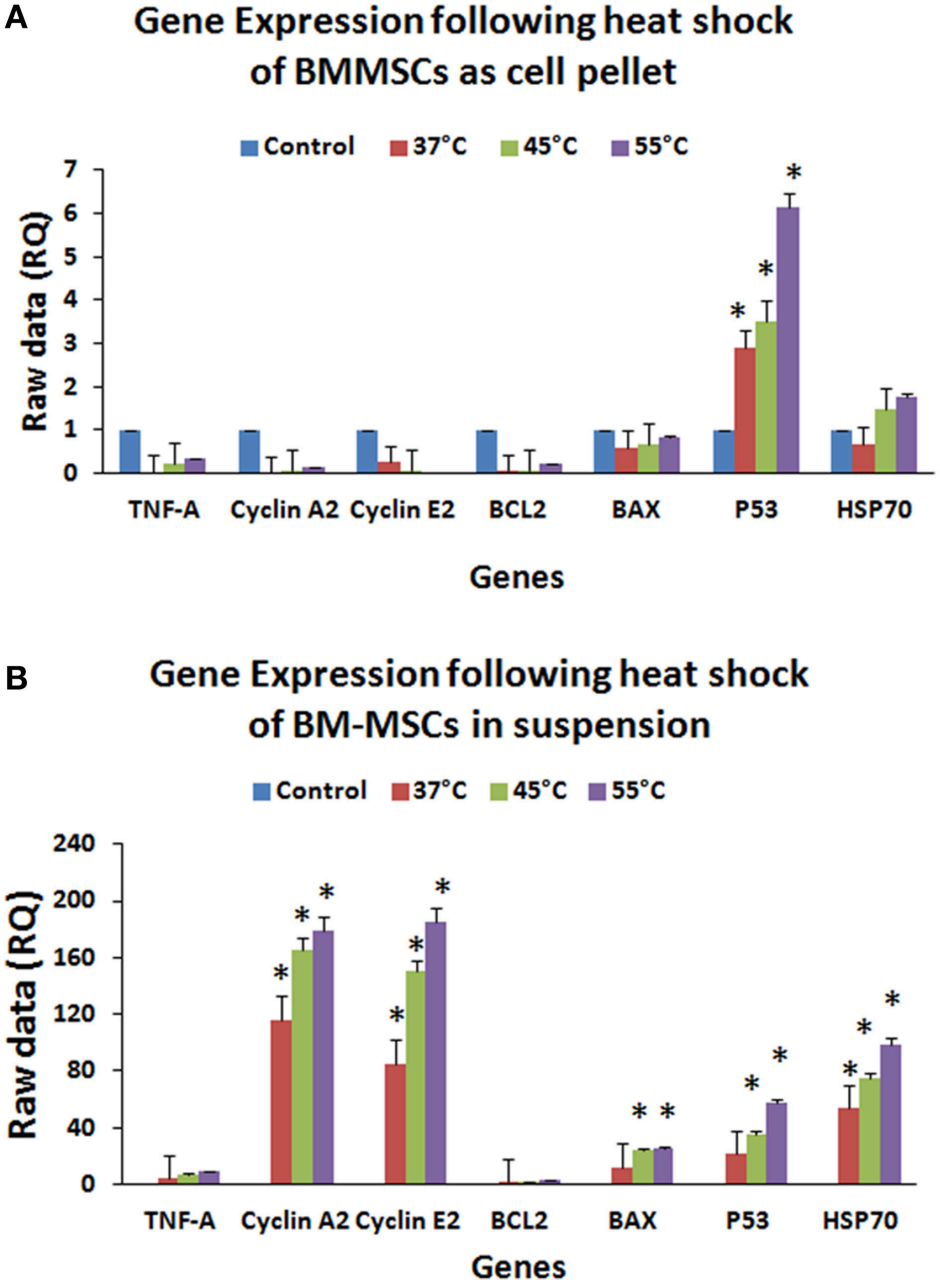
**Gene expression profile (QRT-PCR) of BCL2, BAX, P53, HSP70, TNF-α, CYCLIN A2, and CYCLIN E2 of hBMMSCs exposed to heat shock either as cell pellet (A) or cell suspension (B)**. Upregulation of apoptotic, cell cycle, inflammation and heat shock related genes in hBM-MSCs cell suspension **(B)** compared to cell pellet **(A)** indicate that individual cells **(B)** were easily affected by heat shock compared to cell pellet **(A)**. Data analysis and relative quantitation were performed using the comparative Ct method (ΔΔCt). The differences in gene expression levels were analyzed using student's *t*-test. Asterisks (*) indicate statistical significance at *p* < 0.05.

Gene expression analysis of hBMMSCs exposed to 37, 45, and 55°C for 120 s either as cell suspension or cell pellet demonstrated significant upregulation of the anti-apoptotic, cell cycle and heat shock genes in the cell suspension group compared to the cell pellet group (Figures 7A,B). In the cell suspension group the increase in BAX expression was 12.37-, 23.86-, and 25.18-fold at 37, 45, and 55°C respectively (Figure 7B), while the increase in P53 was 21.24-, 35.77-, 57.36-fold at 37, 45, and 55°C respectively (Figure 7B). Cyclin A2 showed increases of 116.07-, 165.27-, and 179.43-fold in the cell suspension group (Figure 7B), while Cyclin E2 showed increases of 85.13-, 150.51-, and 185.63-fold at 37, 45, and 55°C respectively (Figure 7B). TNF-α expression increased by 3.95-, 7.51-, and 9.41-fold in the cell suspension group (Figure 7B), while the HSP70 expression increased by 53.61-, 75.25-, and 98.11-fold at 37, 45, and 55°C respectively (Figure 7B). In the cell pellet group only P53 showed a significant upregulation and showed increased expression by 2.91-, 3.56-, and 6.16-fold at 37, 45, and 55°C respectively (Figure 7A).

## Discussion

Current pharmacological and surgical therapies for OA provide pain relief and some degree of functional restoration, but a complete cure yet remains elusive. Epidemiological data from the Middle East indicate an increase in the aging and overweight/obese population, both of which are strong risk factors for the development of OA (Loeser et al., 2012). Therefore, it is predicted that OA will grow into a significant health problem in the Middle East as it has become in the West.

Damaged cartilage has a limited self-healing capacity dues to the limited numbers of chondrocytes in mature tissue, poor responses to anabolic factors, impaired extracellular matrix production and lack of blood vessels (Henrotin and Reginster, 1999). Attempts to stimulate intrinsic cartilage repair by bone drilling or microfracturing can promote recruitment of stem cells into the defect area. The presence of MSCs in synovial membrane/fluid is another indication of the potential reparative role that stem cells may play in damaged joint tissue regeneration (Harvanová et al., 2011; Miller et al., 2015). Mesenchymal stem cells from bone marrow, adipose tissue, synovium, umbilical cord and peripheral blood have been used in the management of OA (Richardson et al., 2010; Orth et al., 2014). However, a robust cell type that can be easily harvested and display a high proliferative capacity and chondrogenic potential will be offer significant advantageous for the development of cellular therapies. A recent comparative study on hBMMSCs and adipose tissue derived MSCs (ADMSCs) seeded on Chondro-Gide scaffolds and exposed to chondrogenic medium, identified good proliferation and cartilaginous extracellular matrix deposition with hBMMSCs compared to ADMSCs (Kohli et al., 2015), highlighting the advantage of the former cell type for the development of regenerative medicine approaches.

hBMMSCs derived from OA patients have shown variations with respect to cell proliferation; in some cases cell yields and proliferative indices are high whereas in others they are very poor. Effective expansion of isolated cells is another challenge as the major problem with autologus therapy is the availability of adequate numbers of stem cells. The inoculum density of cells for use in a single 3 mm-4 mm deep subchondral bone drilling site is approximately 2 × 10^6^ cells (Haleem et al., 2010). Therefore, a minimum of 10–12 × 10^6^ cells may be needed for use in 4-6 drill defects. Moreover, these numbers must be achieved in less than 3–4 subcultures to avoid cellular de-differentiation. In the present study, we were able to successfully achieve increased cell yields (approximately about 12 × 10^6^ cells in three to four passages from an initial number of 2 × 10^6^ cells from 5 ml of bone marrow aspirate). Most cells in culture remained viable as shown by the trypan blue viability assay (Figure 4D) and these cells exhibited a short PDT. However, as expected, the target cell expansion was not achieved in some elderly patients. This could be due to the intrinsic senescent nature of the cells as, there appears to be an inverse relationship with cell numbers and aging (Choumerianou et al., 2010).

The derived and expanded hBMMSCs from OA patients were identified to satisfy the minimal criteria set by ISCT. The cells were plastic adherent; showed expression of CD73, CD90, CD105, and CD29 and lacked expression of CD34 and CD45 (Figure 3). These cells also demonstrated tri-lineage differentiation into adipocytes, chondrocytes and osteoblasts (Figure 5). However, in our previous studies of hBMMSCs and human Wharton's Jelly stem cells (hWJSCs) we have demonstrated expression of both osteogenic and chondrogenic related gene and protein expression (Baksh et al., 2007; Gauthaman et al., 2011). In addition, we have shown increased production and secretion of collagen and alkaline phosphatase activity following chondrocytic and osteocytic differentiation respectively, similar to earlier studies obtained by our group and by other investigators (Baksh et al., 2007; Gauthaman et al., 2011; Fong et al., 2012; Brady et al., 2014).

Exposing hBMMSCs to an illuminated arthroscope resulted in decreased metabolic activity of the cells maintained in suspension. However, cell pellets exposed to an illuminated arthroscope demonstrated increased numbers of metabolically active cells (Figures 4B,C). Initially the metabolically active cell numbers increased and remained stable in suspension cultures that were exposed to the illuminated arthroscope for 10 and 20 min. This could be due to the free floating individual cell movements within the medium resulting in a shorter duration of close range exposure of single cells to the illuminated source unlike the outer layer of cells in the pellet, which remained in the same position for the entire duration of exposure. This was reflected initially as lower metabolic activity and/or survival in the cell pellets compared to the cell in suspensions. In contrast, longer exposure of the illuminated arthroscope and the resultant rise in temperature would have affected the suspended cells more adversely compared to most cells in the pellets, expect those that were present in the outer layer. Earlier studies have identified that heat-shock generated by orthopedic procedures leads to death of the transplanted cells. Cell recovery following immediate exposure to 60°C for 30 s to 1 min and subsequent evaluation of proliferation after 12, 24, and 96 h has identified that proliferation decreases and death results almost immediately (mostly by necrosis) (Dolan et al., 2012). However, mild heat shock and exposure of cells to temperatures up to 47°C result in positive osteointegration by osteoprogenitors (Zaffagnini et al., 1996). In contrast to the above studies, the temperature ranged from 27.6 to 37.3°C, indicating that sub-physiological temperature changes or cold-shock can also adversely affect the metabolic activity of transplanted cells. Previous studies have also reported that arthroscopic procedures may be involved with either supra-physiologic or sub-physiologic changes in temperature following low-flow irrigation or cryotherapy (Zaffagnini et al., 1996; Horstman and McLaughlin, 2006). Moreover, the cell aggregates (pellets) were better protected against cold-shock than the cell suspensions.

Differentiation of the BMMSCs following heat shock was not undertaken in the present study, which becomes a constraint to support our other findings. This would have provided additional insights into the functional ability of these cells. Although more in depth studies are needed to understand the mechanisms behind the cold/heat shock related decrease in metabolic activity and/or cell survival, our preliminary findings indicate that in autologous settings the use of hBMMSCs together with chondrocytes may have a protective effect on the transplanted stem cells.

Gene expression studies confirmed that hBMMSCs exposed to increased temperatures resulted in significant upregulation of apoptotic (BAX, P53), cell cycle (Cylcin A2, Cyclin E2) as well as injury and heat shock related (TNF-α, HSP70) genes in cell suspensions compared to cell pellets. Previous studies have reported that increases in temperature ranging from 47 to 70°C as a result of surgical procedures may impair the survival of MSCs (Gill et al., 2007). Thermal stress is known to induce profound changes in protein function and gene expression which in turn activate cellular adaptive mechanisms that help to protect the organism (Leppä and Sistonen, 1997; Hettler et al., 2013). We observed that the heat shock protein HSP70 was increased significantly, which is indicative of an adaptive response of the cells to overcome heat stress and interestingly this was more pronounced in the cell suspension group. Increased expression HSP70 is one of the most important responses following physical and chemical stresses including elevated temperature, exposure to heavy metals, pharmacological agents and bacterial toxins (Sonna et al., 2002; Dolan et al., 2015). Increasing temperatures beyond the threshold limit for a given cell type is known to either activate the apoptotic program or result in cellular necrosis in extreme situations (Creagh et al., 2000; Sonna et al., 2002; Dolan et al., 2015). We also observed that in cell suspensions proapoptotic BAX was increased and the antiapoptotic BCL2 was decreased, indicating that the hBMMSCs were undergoing cell death. Cyclin E2 is involved in G1/S phase of cell cycle progression and cyclin A2 is involved in regulation of G2/M phase of cell cycle. Heat stress following radiation upregulate HSP which in turn augments cell cycle arrest at G1, S, and G2/M phases (Nitta et al., 1997). The increased expression observed for cyclin E2 and Cyclin A2 in cell suspensions may be indicative of an attempt to overcome the heat stress and promote cell survival. The expression level probably decreased as the heat stressed cells in the suspension group underwent necrotic death unlike the pellet group. In addition, P53 is known to influence expression of numerous target genes that control cell cycle, apoptosis, gene instability, senescence following stress (Morselli et al., 2008). Increased expression of P53 was observed following heat stress, and P53 is associated with negative regulation of cell cycle progression (Reisman et al., 2012). If the gene expression of cell cycle inhibitor p21 had been included in the study, it would have provided additional information on the cell proliferation ability following heat shock of hBMMSCs.

The introduction of MSCs into the joint can be achieved either through injection as a cell suspension or through loading onto a scaffold as an implant. Our findings offer caution when the former approach is used since the cells are not encased by a matrix that can protect them from external factors such as heat from the arthroscope. This study suggests that improvizations in surgical methods that avoid generating large amounts of heat may result in more favorable outcomes when applying MSCs in cell suspension to treat an arthritic joint. Alternatively, administration of cells in combination with biodegradable materials as pellets/encapsulations might enhance the survival of transplanted cells.

## Conclusions

hBMMSCs from OA patients have the capacity to expand and actively differentiate. However, they are prone to damage if the method of delivery to the joint is not optimal and is accompanied by the generation of excess heat. Less invasive and more cytoprotective surgical methods that do not generate excess heat are needed to ensure successful therapeutic delivery of MSCs to the joint.

## Author contributions

MAb and MAl are the clinicians and were involved in providing clinical materials/information and intellectual support. GK and MG were involved in conceptualization, intellectual contribution, statistical evaluation and manuscript writing. HA and RK was involved in providing technical assistance with experimental work, manuscript editing and intellectual help. AA, AC, MAQ, and WK were involved in the overall co-ordination of the work, and also reviewed and edited the manuscript. AM contributed to the synthesis and editing of the manuscript.

### Conflict of interest statement

The authors declare that the research was conducted in the absence of any commercial or financial relationships that could be construed as a potential conflict of interest.
